# A Muscle Fatigue Classification Model Based on LSTM and Improved Wavelet Packet Threshold

**DOI:** 10.3390/s21196369

**Published:** 2021-09-24

**Authors:** Junhong Wang, Shaoming Sun, Yining Sun

**Affiliations:** 1 Institute of Intelligent Machines, Hefei Institutes of Physical Science, Chinese Academy of Sciences, Hefei 230031, China; interventionv@foxmail.com (J.W.); ynsun@iim.cas.cn (Y.S.); 2 University of Science and Technology of China, Hefei 230026, China

**Keywords:** surface electromyography, wavelet packet, muscle fatigue, long short-term memory

## Abstract

Previous studies have used the anaerobic threshold (AT) to non-invasively predict muscle fatigue. This study proposes a novel method for the automatic classification of muscle fatigue based on surface electromyography (sEMG). The sEMG data were acquired from 20 participants during an incremental test on a cycle ergometer using sEMG sensors placed on the vastus rectus femoris (RF), vastus lateralis (VL), vastus medialis (VM), and gastrocnemius (GA) muscles of the left leg. The ventilation volume (VE), oxygen uptake (VO_2_), and carbon dioxide production (VCO_2_) data of each participant were collected during the test. Then, we extracted the time-domain and frequency-domain features of the sEMG signal denoised by the improved wavelet packet threshold denoising algorithm. In this study, we propose a new muscle fatigue recognition model based on the long short-term memory (LSTM) network. The LSTM network was trained to classify muscle fatigue using sEMG signal features. The results showed that the improved wavelet packet threshold function has better performance in denoising sEMG signals than hard threshold and soft threshold functions. The classification performance of the muscle fatigue recognition model proposed in this paper is better than that of CNN (convolutional neural network), SVM (support vector machine), and the classification models proposed by other scholars. The best performance of the LSTM network was achieved with 70% training, 10% validation, and 20% testing rates. Generally, the proposed model can be used to monitor muscle fatigue.

## 1. Introduction

The neuromuscular system consists of the nervous system and the muscular system. The main function of the human muscular system is to provide the energy needed by the human body to perform various actions. Exercise-induced muscle fatigue is a physiological phenomenon in which the maximum voluntary contraction (MVC) capacity and output power of the muscle decreases. The cause of exercise-induced muscle fatigue is the accumulation of metabolites (lactic acid, hydrogen ion, inorganic phosphate) in the human blood during exercise [1]. The risk of sports injuries increases with muscle fatigue. Therefore, the precise positioning of fatigued muscles is the basis for relieving and curing muscle fatigue and has important sports medicine significance. Electromyography (EMG) is an electrical signal generated by skeletal muscles when they contract spontaneously [2]. EMG signal analysis can provide continuous measurement of the muscle’s state during the continuous fatigue contraction process, which is different from the subjective typical assessment that usually indicates that the subject can no longer perform the test [3]. The surface electromyography signal (sEMG) is a comprehensive effect of superficial muscle EMG and nerve trunk electrical activity on the skin surface. sEMG can reflect neuromuscular activity to a certain extent, and it has the advantages of non-invasiveness and simple operation [4]. The analysis of the sEMG signal is performed by a trained neurologist. When there are not enough experts to meet the needs of fatigue warning, it is very important to use deep learning technology to realize neuromuscular fatigue detection and classification on the basis of EMG signal processing.

However, the sEMG signals are affected by many confounding factors, including the following: the shape of the volume conductor; thickness of the subcutaneous tissue layers; distribution and size of motor unit areas in muscles; changes in transmembrane action potential; conductivities of the tissues; electrode position; detection system; etc. [5]. Wavelet and wavelet packet transform are a multiresolution decomposition method that can be used to analyze signals and images denoising. Both wavelet transform and wavelet packet transform are multiresolution decomposition methods, which can characterize local features in the time domain and frequency domain, and they are usually used to analyze signals and image denoising [6]. Li et al. analyzed and compared several different wavelet threshold denoising algorithms, and they proposed a new wavelet threshold denoising algorithm to effectively eliminate noise interference under strong noise background and preserve signal details [7]. Traditional threshold functions include hard threshold and soft threshold functions, which have some deficiencies in signal de-noising. To solve these shortcomings, Zhang et al. proposed an improved wavelet threshold method to denoise MRI images [8]. The results show that the improved wavelet threshold method in denoising MRI images has better performance than the traditional wavelet threshold function.

When the local muscle is fatigued, the sEMG signal of the muscle will change in the time domain and the frequency domain [9]. Many scholars have studied muscle fatigue recognition models that can identify muscle fatigue states by detecting changes in sEMG signals. Latasa et al. detected the EMG signals of cyclists in an incremental continuous cycling test and used a multi-segment linear regression algorithm to study the aerobic-anaerobic threshold transition [3]. Martinez et al. recorded the subjects’ sEMG signals in the fatigue state of the vastus lateralis in the incremental cycling test and analyzed the correlation between muscle fiber conduction velocity (MFCV), instantaneous mean frequency (iMNF), normalized root mean square (RMS), and muscle fatigue state [10]. Subasi and Kiymik used independent component analysis (ICA) to process the surface EMG signal of the biceps brachii, and they used artificial neural network (ANN) to classify muscle fatigue [11]. M-wave normalization of the sEMG signal can describe the activity amplitudes in the state of muscle fatigue during repeated sprinting [12]. Wu et al. proposed a BFA–Gaussian support vector machine (GSVCM) model to improve the accuracy of muscle fatigue recognition [13]. Hussain and Mamun utilized different wavelet functions (WFs) to analyze the EMG signal of the right rectus femoris muscle to identify the fatigue state of the right rectus femoris muscle during walking [14]. Consequently, they used these functions for accurate automated muscle fatigue classification and systematically processing the EMG signal based on deep learning and machine learning methods.

This paper researches a new muscle fatigue state recognition model. First of all, we present a new wavelet packet threshold function denoising method to denoise sEMG signals. The signal-to-noise ratio (SNR) and root mean square error (RMSE) parameters of this method, hard threshold, and soft threshold denoising are compared. Secondly, we describe the time-domain and frequency-domain feature extraction applied in the sEMG signals classification process. We divided the sEMG signals into two groups: non-fatigue and fatigue, based on the anaerobic threshold (AT). Finally, the long and short-term memory (LSTM) network was used to identify muscle fatigue and compared with other classification algorithms in terms of classification performance.

## 2. Materials and Methods

### 2.1. Participants

Experimental participants: Twenty healthy males (*n* = 20; Age range: 22–33 years; Height: 1.79 ± 0.09 m; Weight: 65.5 ± 6.6 kg) were recruited to participate in this experiment. All participants were screened and free from cardiovascular, neuromuscular, and metabolic diseases. Before the test, all participants voluntarily participated in the test, fully understood the purpose, details, methods, and potential test risks of the experiment, and signed an informed consent form. They did not engage in vigorous exercise and did not consume caffeine, nicotine, and alcohol within 48 h before testing. The study was conducted in accordance with the Declaration of Helsinki and the National law of China.

### 2.2. Instrumentation

To ensure that the power is accurately regulated, the exercise test wase performed in the Lode Corival cpet cycle ergometer. The Ag–AgCl surface electrodes were arranged in bipolar configuration (20 mm center-to-center, 1 cm in diameter), and a Noraxon Ultium sEMG sensor were used to record the sEMG signal during the experiment. The sample frequency of the Noraxon Ultium sEMG sensors is 2000 Hz. We use Noraxon MR3 software to analyze the collected sEMG signals offline. The Ultima GX system was used to measure the participants’ ventilation (VE), oxygen uptake (VO_2_), and carbon dioxide production (VCO_2_) during the exercise test. We calibrated all the sensors and experimental equipment before the experiment.

The experimental data analysis was carried out on a workstation with an Intel Core^TM^ i7-9700 and 16 GB memory card. The preparation of the simulation programs was carried out on MATLAB 2020a.

### 2.3. Procedure

**Figure 1:**
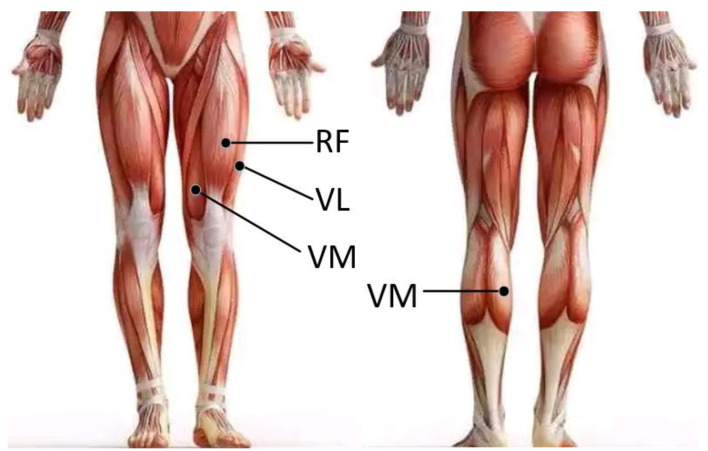
Description of muscle location on the left leg and sEMG electrode placement; GA, RF, VM, and VL muscles.

Before the experiment, in order to reduce the impedance, it is necessary to shave the excess hair of the skin of the participant’s legs and wipe it with medical alcohol. Then, we placed sEMG sensors on the appropriate position of the vastus rectus femoris (RF), vastus lateralis (VL), vastus medialis (VM), and gastrocnemius (GA) muscles according to the SENIAM (Surface EMG for Non-Invasive Assessment of Muscles) guidelines [15]. In order for participants not to be affected during the execution of the procedure, the sports bandages were used to fix the sEMG sensors. The position of the sEMG sensors on the left leg is shown in Figure 1.

**Figure 2:**
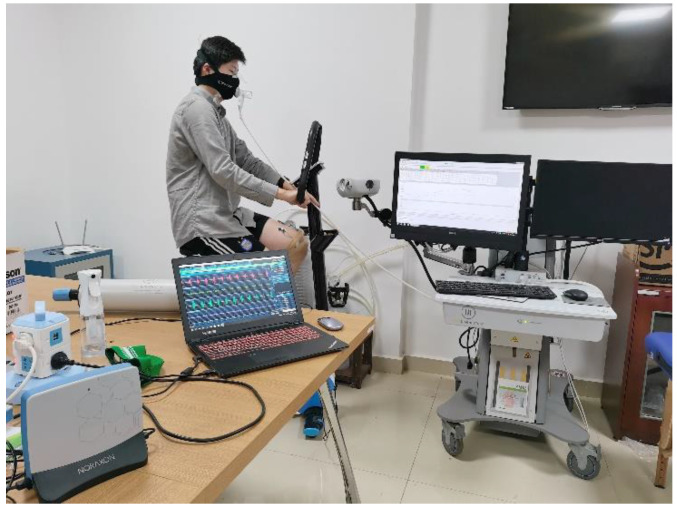
Representative participant performs exercise test.

Before the test, participants could use equipment and procedures proficiently. After a 3 min warm-up on a cycle ergometer (Lode Corival cpet), each of the participants performed an incremental protocol in a temperate environment (25–28 ℃), starting at a 100 W initial workload with increases of 25 W every 1 min. The participants were instructed to maintain a pedaling rate within the range of 70–75 r/min throughout the test [16,17]. During the test, we strongly encouraged each participant to provide a maximal effort. The test was terminated when the participant was unable to maintain a pedaling rate above 70 r/min due to volitional exhaustion. The participant performs the test as shown in Figure 2.

After the test, we use the V-slope method to calculate the AT based on analyzing the slopes of VO_2_ and VCO_2_ volume curves [18]. We divide the sEMG signals into fatigue and non-fatigued status at the time corresponding to the AT.

### 2.4. Wavelet Packet Threshold Denoising

The wavelet packet threshold denoising algorithm uses the multi-scale characteristics of wavelet packet analysis to decompose the signal by wavelet packet. The threshold function determines whether the wavelet signals of different layers are noise signals according to the threshold. The wavelet coefficients related to noise become corresponding appropriate values according to a different number of layers. In the threshold denoising method based on wavelet transform, the selection of wavelet basis, the number of wavelet decomposition layers, the threshold, and the threshold function have a great influence on the effect of wavelet threshold denoising [19]. According to previous research, the Daubechies (Db) wavelet family has the most suitable wavelet functions for sEMG signals denoising analysis [20,21]. Therefore, this paper uses the db45 wavelet function for the wavelet packet decomposition. This chapter will mainly describe the optimal wavelet packet decomposition layer algorithm and the improved wavelet threshold function.

#### 2.4.1. Best Tree Wavelet Packet Analysis

**Figure 3:**
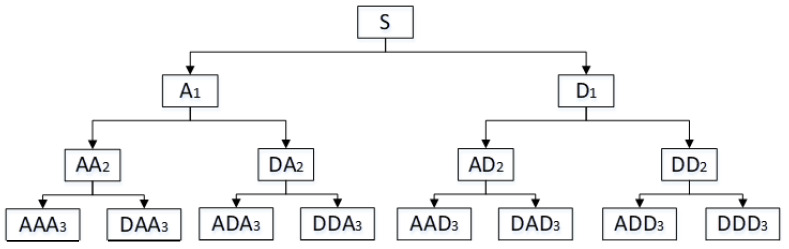
Schematic diagram of wavelet packet decomposition tree at level 3.

**Figure 4:**
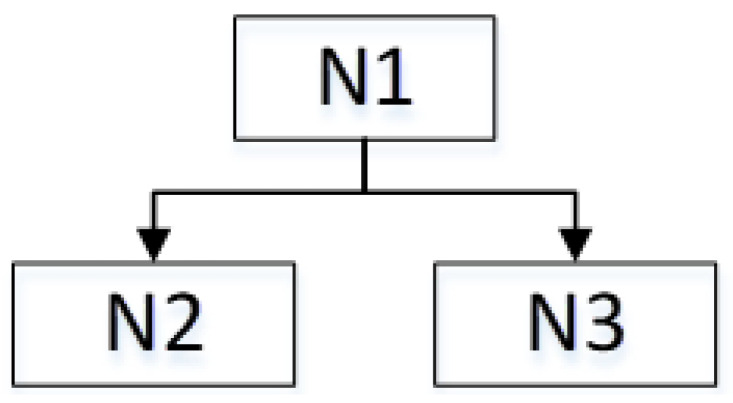
Best tree decomposition principle.

The wavelet packet decomposes the sEMG signals to obtain high-frequency coefficients and low-frequency coefficients. Both the high-frequency coefficient and the low-frequency coefficient are decomposed by analogy as the input signal of the next stage until the decomposition reaches the set number of layers. The complete binary tree is produced as shown in Figure 3. Before the decomposition of the wavelet packet, it is necessary to find the best tree of the wavelet packet. Starting with the root node, the best tree is calculated using Shannon entropy. The Shannon entropy calculation method is as follows.

(1) $$E(s_{i})=-\sum_{i\in Z}s_{i}^{2}log(s_{i}^{2})$$

where $s_{i}$ is the wavelet packet coefficient sequence. The following method is used to calculate the best tree. A node N1 is split into two nodes N2 and N3 if and only if the sum of the entropy of N2 and N3 is lower than the entropy of N1; otherwise, node N1 will not be decomposed [22]. This is a local criterion based on the information available at node N1, as shown in Figure 4. It is calculated by the Shannon entropy criterion in which the decomposition of four layers is most suitable in the sEMG signals’ wavelet packet decomposition.

#### 2.4.2. Threshold Function

The threshold function is essential for sEMG signal denoising analysis. The hard threshold and soft threshold functions proposed by Donoho [23] are extensively used. The hard threshold and soft threshold functions are expressed as Equations (2) and (3), respectively.

The hard threshold function is calculated as follows:

(2) $${\hat{w}}_{j,k}=\left\{ \begin{array}{l} w_{j,k},\;\left|w_{j,k}\right|\geq \lambda \\ \;\;\;\;0,\;\left|w_{j,k}\right|<\lambda \end{array}\right.\;.$$

The soft threshold function is expressed as follows:

(3) $${\hat{w}}_{j,k}=\left\{ \begin{array}{l} \mathrm{sign}(w_{j,k})(\left|w_{j,k}\right|-\lambda ),\;\left|w_{j,k}\right|\geq \lambda \\ \;0,\;\;\;\;\;\;\;\;\;\;\;\;\;\;\;\;\;\;\;\;\;\;\;\;\;\;\left|w_{j,k}\right|<\lambda \end{array}\right.\;\;.$$

Although the above-mentioned traditional signal denoising methods have some effects in practical applications, it is undeniable that they still have shortcomings. The disadvantage of the hard threshold function itself is discontinuous at threshold λ, and the wavelet coefficients ($w_{j,k}$) larger than the threshold λ are not processed, and the $w_{j,k}$ smaller than the threshold λ are set to zero, which will cause oscillations in the reconstruction of $w_{j,k}$. Although the soft threshold function is continuous and derivable at the threshold λ, the wavelet coefficients processed by Equation (3) deviate from the actual wavelet coefficients. In order to compensate for the deficiencies of the above-mentioned threshold function, we proposed the improved threshold function as follows:

(4) $${\hat{w}}_{j,k}=\left\{ \begin{array}{l} w_{j,k}-\frac{m\cdot w_{j,k}}{1+log(\frac{\left|w_{j,k}\right|}{\lambda })},\;\;\;\;\left|w_{j,k}\right|\geq \lambda \\ \frac{(1-m)sign(w_{j,k}){\left|w_{j,k}\right|}^{k+1}}{(1-log(\frac{\left|w_{j,k}\right|}{\lambda })\lambda^{k}},\;\;\left|w_{j,k}\right|<\lambda \end{array}\right.\;.$$

When $\left|w_{j,k}\right|\to \lambda^{+}$, Equation (4) can be written as:

(5) $$\underset{\left|w_{j,k}\right|\to \lambda^{+}}{\lim}(w_{j,k}-\frac{m\cdot w_{j,k}}{1+log(\frac{\left|w_{j,k}\right|}{\lambda })})=(1-m)\lambda .$$

When $\left|w_{j,k}\right|\to \lambda^{-}$, Equation (4) can be written as:

(6) $$\underset{\left|w_{j,k}\right|\to \lambda^{-}}{\lim}(\frac{(1-m)sign(w_{j,k}){\left|w_{j,k}\right|}^{k+1}}{(1-log(\frac{\left|w_{j,k}\right|}{\lambda })\lambda^{k}})=(1-m)\lambda .$$

In Equations (2)–(4), $w_{j,k}$ is the wavelet coefficient, and *λ* is the threshold. Parameters *k* and *m* in Equation (4) include the adjustment parameters (k∈N,m∈(0,1)). The improved threshold function is not only continuous at the threshold but also high-order differentiable. The denoising effect of the wavelet packet threshold denoising algorithm is closely related to the choice of threshold and threshold function [24]. In recent years, the following four threshold estimation criteria have been widely used: fixed threshold estimation (Sqtwolog), maximal minimum threshold estimation (Minimaxi), unbiased risk estimation (Rigsure), and heuristic threshold estimation (Heursure). In this paper, we select the Heursure estimation method to calculate the threshold *λ*. The algorithm flow is as follows:

1. Square each element in the vector W, and then sort in ascending order to obtain a new vector $\overset{\wedge }{W}$ ($\overset{\wedge }{W}(1)$, $\overset{\wedge }{W}(2)$, …, $\overset{\wedge }{W}(N)$). The length of the vector W is the integer N.
2. The threshold is the square root of the *i*-th element of the vector $\overset{\wedge }{W}$; then, the risk algorithm is as follows [25]:
  (7) $$risk(i)=\frac{N-2k+\sum_{j=1}^{i}\overset{\wedge }{W}(j)+(N-i+1)*\overset{\wedge }{W}(N-i+1)\;}{N}\;\;\;\;\;i=1,2,\ldots \;,N.$$
3. Calculate the *i* value corresponding to the minimum risk (*i*), as shown in Equation (8). The threshold λ calculation method is shown in Equation (9).
  (8) $$\hat{i}=\underset{i}{\arg\min}(risk(i))$$
  (9) $$\lambda =\sqrt{\overset{\wedge }{W}(\hat{i})}$$

The procedure of the wavelet packet threshold denoising algorithm is as follows [26]:

1. Select the appropriate wavelet function, and perform the wavelet packet decomposition calculation according to the best tree of the wavelet packet;
2. A threshold value is selected for the wavelet packet coefficients of each decomposition scale. Select the appropriate threshold function to process the wavelet packet coefficients $w_{j,k}$;
3. The processed wavelet packet coefficients ${\hat{w}}_{j,k}$ are reconstructed by inverse wavelet packet transformation. The denoised signals are obtained.

The objective evaluation of the wavelet packet threshold denoising algorithm is described by the signal-to-noise ratio (SNR) and mean square error (MSE). The calculation methods of SNR and RMSE are shown in Equations (10) and (11).

(10) $$SNR=10*\mathrm{lg}\left[\frac{\sum_{n}{\hat{x}}^{2}(n)}{\sum_{n}{\left[\hat{x}(n)-x(n)\right]}^{\;2}}\right]$$

(11) $$RMSE=\sqrt{\frac{1}{n}\sum_{n}{\left[\hat{x}(n)-x(n)\right]}^{\;2}}$$

In Equations (10) and (11), $\hat{x}(n)$ is the original signal, x(n) is the signal after denoising, and n is the signal length.

### 2.5. Feature Extraction

The sEMG signal is a 1D time-series signal of the neuromuscular system that is recorded on the skin surface. The analysis of sEMG concentrated on two main fields: the frequency domain and the time domain [27]. In this study, we choose two time-domain features, Root Mean Square (RMS) and Integrated Electromyogram (IEMG), to describe the changes in the amplitude of the sEMG signals. The mathematical expressions of RMS and IEMG are expressed as:

(12) $$\mathrm{IEMG}=\int_{t}^{t+T}\left|x(t)\right|dt$$

(13) $$\mathrm{RMS}=\sqrt{\frac{1}{T}\int_{t}^{t+T}x^{2}(t)dt}.$$

The frequency-domain feature is extracted from the Fourier transform of the signal. We choose median frequency (MF) and mean power frequency (MPF) because of their abilities to reflect the fatigue-caused frequency changes of sEMG [28,29,30]. The mathematical expressions of MF and MPF are expressed as:

(14) $$\int_{f_{1}}^{\mathrm{MF}}P(f)df=\int_{\mathrm{MF}}^{f_{2}}P(f)df\;$$

(15) $$\mathrm{MPF}=\frac{\int_{f_{1}}^{f_{2}}f*P(f)df}{\int_{f_{1}}^{f_{2}}P(f)df}\;.$$

In Equations (14) and (15), *f*_1_ and *f*_2_ determine the bandwidth, and *P*(*f*) is the power spectral density (PSD) of the sEMG signals. *P*(*f*) is expressed as:

(16) $$P(f)=\frac{{\left|x(f)\right|}^{2}}{L}.$$

In the above equations, *L* is the signal length, and *x*(*f*) is the sEMG signals in the frequency domain. The RMS, IEMG, MF, and MPF are extracted from the denoised sEMG signals by a moving window of 2 s. In order to construct a muscle fatigue feature data set, we divide the sEMG feature data into fatigue and non-fatigued sEMG data according to the time point of the AT. The V-Slope method was introduced to calculate the AT in the fatigue test.

### 2.6. Fatigue Recognition Model

**Figure 5:**
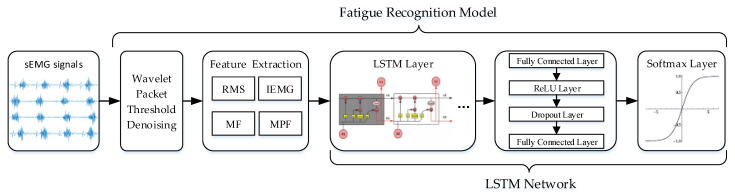
Representation of the proposed method.

The fatigue recognition model classifies the sEMG signal of muscle fatigue status and muscle non-fatigue status and was constructed based on LSTM networks. The proposed method is presented in Figure 5. The LSTM network is a modified version of recurrent neural network (RNN). The control system of the LSTM unit consists of input, output and forget gates. The internal state $c_{t}$ of the LSTM network records the historical information up to the current moment, and the three gates control the information transmission path [31]. The forget gate $f_{t}$ controls the amount of information that needs to be forgotten in the internal state $c_{t-1}$ at the previous moment, and the activation state of the forget gate is computed as shown in Equation (17).

(17) $$f_{t}=\sigma (W_{f}x_{t}+U_{f}h_{t-1}+b_{f})$$

where $x_{t}$ is the current input vector of the LSTM unit, $h_{t-1}$ is the output of the previous LSTM unit, σ is the logistic sigmoid function, $W_{f}$ is the weight vector, and $b_{f}$ is the biased vector. The input gate $i_{t}$ determines the amount of information stored in the current candidate state, and the calculation method is shown in Equations (18) and (19).

(18) $$i_{t}=\sigma (W_{i}x_{t}+U_{i}h_{t-1}+b_{i})\;$$

(19) $$c_{t}=f_{t}⊗c_{t-1}+i_{t}⊗\tanh(W_{c}x_{t}+U_{c}h_{t-1}+b_{c})$$

The data and information of the current LSTM unit are conveyed to the output gate, and the output calculations are shown in Equations (20) and (21).

(20) $$o_{t}=\sigma (W_{o}x_{t}+U_{o}h_{t-1}+b_{o})\;$$

(21) $$h_{t}=o_{t}⊗\tanh(c_{t})$$

The LSTM network used in this study consists of an LSTM layer, fully connected layer, rectified linear unit (ReLU) layer, dropout layer, and softmax layer. The fully connected layer connects all neurons of the previous and next layers. The dropout layer can make a certain neuron activation value stop working with a certain probability, which can make the model more generalized and prevent overfitting. Finally, the activation function of the softmax layer was used to classify the muscle state. The LSTM network used the initial hyperparameter configuration shown in Table 1. We selected the Stochastic Momentum Gradient Descent (SGDM) algorithm to optimize the learnable parameters. During the LSTM network training process, these hyperparameters were reset many times until the optimal configuration was reached.

We constructed muscle fatigue recognition models based on SVM (support vector machine) and CNN (convolutional neural network) respectively and then compared the LSTM muscle fatigue recognition models. The kernel function has an important impact on the classification performance of the SVM. Numerous applications indicate that the Gaussian kernel function has good learning capability, so the SVM used in this study uses the Gaussian kernel function. The CNN network consists of 5 layers: an input layer, two convolutional layers, and two fully connected layers. The cross-entropy loss function is used to conduct back-propagation training based on the error. The initial learning rate is 0.1.

### 2.7. Evaluation of the Proposed Model

**Figure 6:**
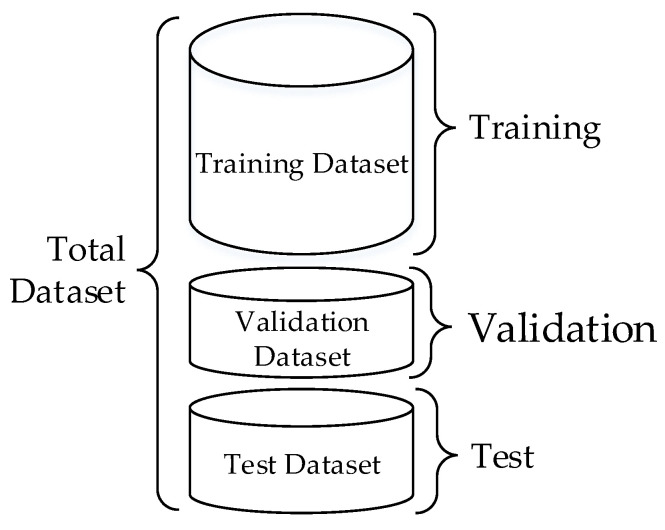
Holdout method for training, validation, and testing.

In order to ensure the generalization ability of the model, the muscle fatigue recognition model needs to be trained, verified, and tested on an independent data set [32]. The verification method selected in this article is Holdout. This method consists of dividing the data into three independents subsets: training, validation, and test. The premise is that the ratio of fatigue data to non-fatigued data in each subset is approximately equal to the ratio of fatigue data to non-fatigued data in the total sEMG data set. The training data set was used for LSTM network training. The validation data set was used to evaluate performance during training through accurate measurements and error. The test data set was used for a final evaluation of the predictions performed by the model. Three datasets were independent, as Figure 6 shows. The dataset division mode was divided into 3 types: (a) 70%, 10%, 20% (training, validation, testing); (b) 60%, 10%, 30% (training, validation, testing); (c) 50%, 10%, 40% (training, validation, testing).

We select four indicators of accuracy (Acc), sensitivity (Sn), specificity (Sp), and precision (Pr) to evaluate the performance of the model. The calculation methods of the above indicators are shown in Equations (22)–(25), respectively.

(22) $$Acc=\frac{TF+TN}{TF+TN+FF+FN}$$

(23) $$Sn=\frac{TF}{TF+FN}$$

(24) $$Sp=\frac{TN}{TN+FF}$$

(25) $$Pr=\frac{TF}{TF+FF}$$

The parameters *TP*, *TN*, *FF*, and *FN* in Equations (22)–(25) are true fatigue (*TF*), true non-fatigue (*TN*), false fatigue (*FF*), and false non-fatigue (*FN*).

## 3. Results

### 3.1. Evaluation of Enoising Algorithm Performance

In order to illustrate the effectiveness of the wavelet packet threshold algorithm in denoising sEMG signals, the traditional threshold function is compared with the improved threshold function proposed. Our sEMG data were obtained from the MIT-BIH Normal Sinus Rhythm Database (Accessed date: 2 July 2021. https://physionet.org/content/emgdb/1.0.0/). A total of 26,860 points were selected after 6 s of the first channel of the emg_healthy signal, and they were used in this study after adding Gaussian white noise.

**Figure 7:**
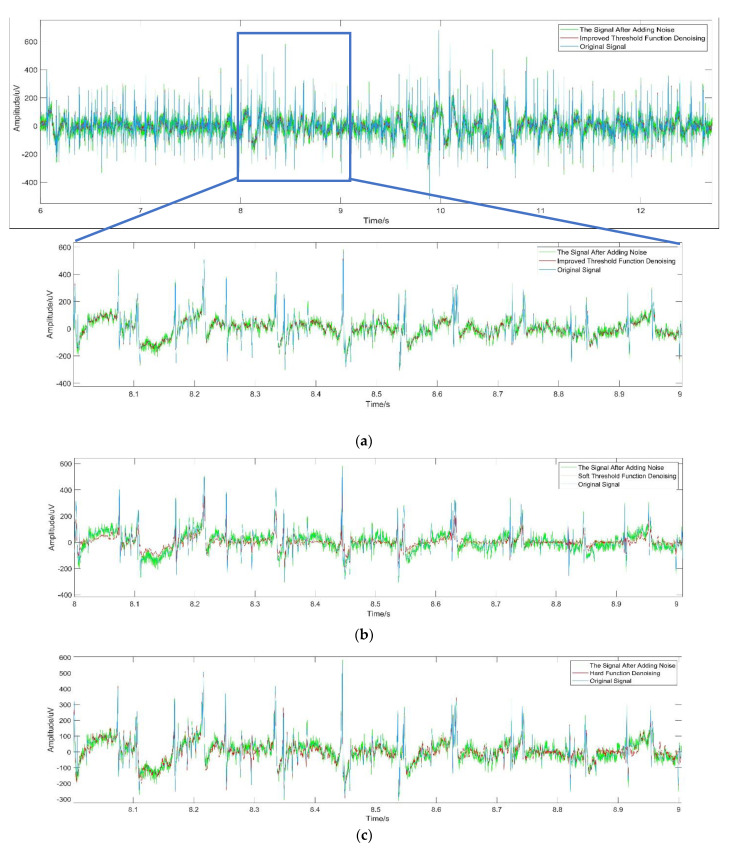
(**a**) Improved threshold function denoising for the EMG signal; (**b**) Soft threshold function denoising for the EMG signal; (**c**) Hard threshold function denoising for the EMG signal.

The denoising effect comparison between the improved wavelet packet threshold function and the traditional threshold function is shown in Figure 7. It can be seen from the waveform before and after denoising that the reconstructed waveform after the denoising of the wavelet packet-improved threshold function is relatively smoother, and its amplitude is almost consistent with the original signal, which retains useful information. The denoising performance is shown in Table 2; it can be seen that the SNR of the sEMG signals after the improved threshold function denoising is nearly 49.34% and 56.98% higher than that of the sEMG signals after the hard and soft threshold functions, respectively. The RMSE values of the sEMG signals denoised by the improved threshold function are 51.01% and 16.34% lower than the RMSE of the hard threshold function and soft threshold function, respectively.

**Figure 8:**
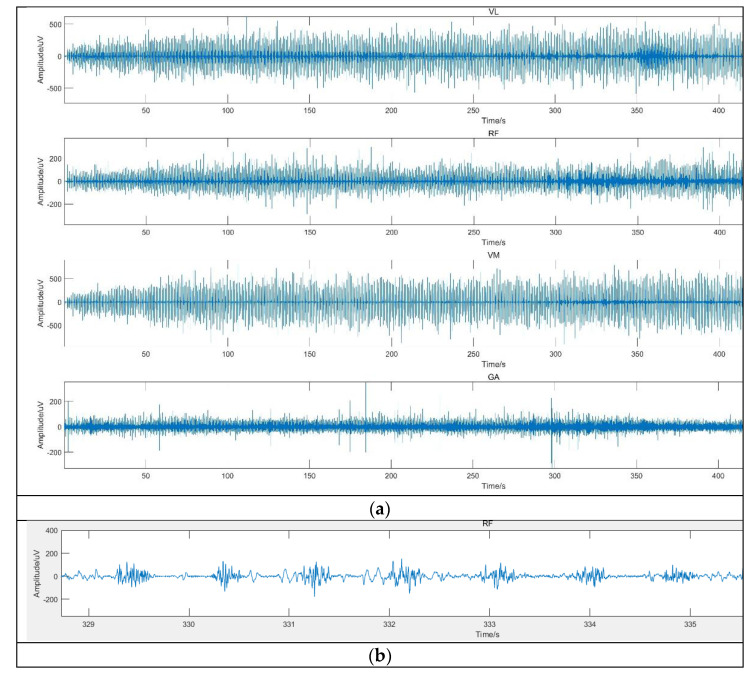
(**a**) The sEMG signals of VL, RF, VM, and GA denoised by improved wavelet packet threshold function; (**b**) sEMG signal of RF after local amplification.

The sEMG signals denoised by the wavelet packet improved threshold algorithm is shown in Figure 8. Based on these signals, time and frequency-domain feature data would be extracted to construct a training–test dataset.

### 3.2. Fatigue Status Recognition

**Figure 9:**
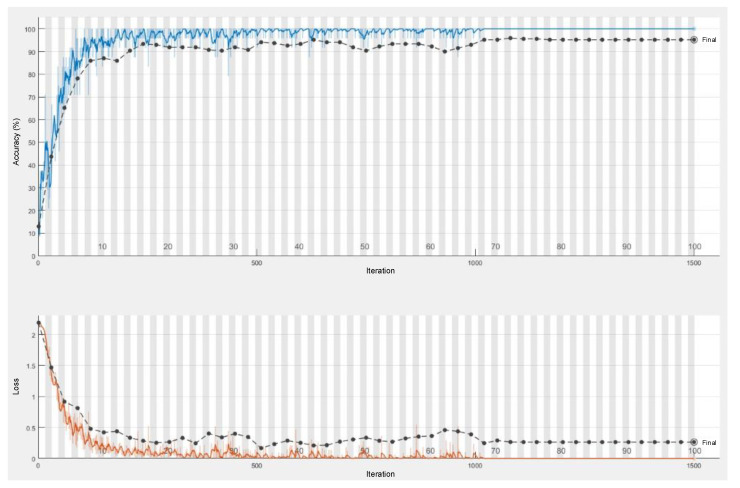
The graphs of accuracy and loss values for the LSTM training and validation.

The graphs of training, validation, testing (70%, 10%, 20%) accuracies, and training testing loss values are given in Figure 9. The training and testing time was 1702 s.

**Figure 10:**
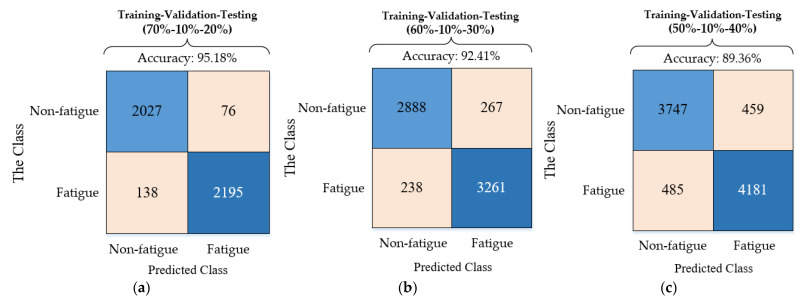
Confusion matrix results for sEMG feature data and the rates of training–validation–testing.

The performance criteria results, which consist of accuracy, sensitivity, specificity, and precision, are shown in Table 3. As can be seen from Table 3, in the process of using the LSTM network to perform fatigue classification on the sEMG feature dataset, the best classification performance that was obtained with the training data, the verification data, and the test data accounted for 70%, 10%, and 20% of the entire data set, respectively. Moreover, the best classification accuracy of CNN and SVM that was also obtained with the training data, the verification data, and the test data accounted for 70%, 10%, and 20% of the entire data set, respectively. In Table 3, the worst result in the evaluation of muscle fatigue status recognition is the accuracy standard of 0.8569, which is obtained from the training–validation–testing rate of 50% 10% 40% and CNN. For 70%, 10%, 20% rates of training–validation–testing, the confusion matrix results of LSTM, SVM, and CNN are shown in Figure 10. The performance of the LSTM model with the sEMG signals denoised by the improved threshold function as input is better than the LSTM model with the sEMG signals denoised by the hard and soft threshold denoising as input, as shown in Table 4. For instance, the classification accuracy of the combination of the improved wavelet threshold function and the LSTM model is 6.47% and 3.85% higher than the hard threshold function and the soft threshold function, respectively.

## 4. Discussion

In this section, the proposed methods and common methods will be discussed in terms of denoising performance and classification performance. The original sEMG signal was almost submerged by noise after adding Gaussian noise. The sEMG signal denoised by the hard threshold and soft threshold function still retains considerable noises. By comparing the images (Figure 7 and Figure 8) and denoising performance (Table 2), it can be seen that the improved threshold function denoising method is more suitable for surface EMG signal denoising than the other two threshold functions. The signal denoised by the improved threshold function has better performance than the signal denoised by the hard threshold and soft threshold function in approximating the original signal.

The classification performancea of these methods are given in Table 4 Wu et al. [13] proposed a novel bacterial foraging algorithm (BFA)–Gaussian support vector classifier machine (GSVCM) model to improve the muscle fatigue classification accuracy. The RMS, IEMG, MPF, MF, and mean instantaneous frequency (MIF) features of the sEMG signals were extracted to evaluate the fatigue status during muscle contraction. With the training–testing rate of 80% 20%, the GSVCM model achieves the best accuracy of 93.94%. Khan et al. [33] constructed three separate random forest models to classify muscle fatigue status based on eight sEMG features, with the best accuracy 87%. A CNN-SVM algorithm was proposed to identify muscle fatigue status [33]. The RMS, IEMG, MPF, MF, and BSE features of the sEMG signals are extracted as the input dataset of the algorithm, and the best recognition accuracy is 86%.

This research proposes a new muscle fatigue recognition model based on LSTM, which is learned from scratch. In the preprocessing stage, the collected sEMG signal was processed by the wavelet packet threshold function denoising algorithm, and then the time-domain and frequency-domain features of the signal were extracted as the input of the LSTM algorithm. As shown in Table 5, the wavelet packet threshold function denoising algorithm improves the performance of the classification algorithm, and the improved wavelet threshold function denoising algorithm has the best effect. With the same dataset as input, the LSTM algorithm performs better than CNN, SVM, and BFA–GSVCM in the accuracy of muscle fatigue recognition. The model proposed in this paper was trained, validated, and tested based on three different dataset ratios. The best accuracy of the model was obtained at type (a) 70%, 10%, 20% training, validation, testing. This indicates that the classification performance of the proposed model will improve with the increase in sEMG training data. However, the computation time for training process of the new muscle fatigue classification model is relatively long.

In addition, the model can be applied to the automatic detection of leg muscle fatigue during exercises other than cyclic resistance exercises. This model can be used as an auxiliary tool for coaches to monitor the muscle state of cyclists and long-distance runners during training. In the future, this model can be used to detect the state of muscles in other parts of the human body during exercise.

## 5. Conclusions

In this study, we analyzed the shortcomings of the traditional threshold function in the denoising of sEMG signal and proposed an improved threshold function. We compared the classification performance of LSTM with CNN, SVM, and other classification algorithms. In conclusion, the muscle fatigue recognition model constructed based on the improved wavelet packet threshold function denoising algorithm and LSTM network has excellent performance in the denoising of sEMG signal and the classification of muscle fatigue. The experimental results prove that the improved wavelet packet threshold function denoising algorithm is significantly better than the hard threshold and soft threshold functions in denoising the EMG signal. When the dataset was divided into training–verification–test 70% 10% 20%, the LSTM network achieved the best performance, including accuracy, sensitivity, specificity, and precision. Compared with other classification algorithms, the LSTM network achieved the best performance, including accuracy, sensitivity, and precision. However, we plan to improve the accuracy of the model in terms of feature extraction and algorithm optimization.

## Figures and Tables

**Table 1:** LSTM configuration.

Hyperparameter	Value
LSTM units	100
Layers	5
Loss function	RTRL
Optimizer	Adam
Activation	ReLU
Batch size	70
Initial learning rate	0.001

**Table 2:** Denoising results of the three threshold functions.

Threshold Function	SNR	RMSE
Hard Threshold Function	13.274	15.892
Soft Threshold Function	12.627	9.307
Improved Threshold Function	19.823	7.786

**Table 3:** Performance criteria for training–validation–testing rate.

Training–Validation–Testing Rates (%)	Methods	Acc	Sn	Sp	Pr
70–10–20	LSTM	0.9518	0.9408	0.9638	0.9665
60–10–30	LSTM	0.9241	0.9319	0.9153	0.9243
50–10–40	LSTM	0.8936	0.8960	0.8908	0.9010
70–10–20	CNN	0.9272	0.9124	0.9081	0.9279
60–10–30	CNN	0.9013	0.8928	0.8853	0.8976
50–10–40	CNN	0.8569	0.8398	0.8426	0.8578
70–10–20	SVM	0.9030	0.8733	0.8916	0.9042
60–10–30	SVM	0.8874	0.8805	0.8919	0.8882
50–10–40	SVM	0.8611	0.8673	0.8590	0.8717

**Table 4:** The classification performance of the proposed method.

Methods	Number of Feature	Dataset	Acc(%)
Random Forest Model [33]	8	Private	87.00
BFA–GSVCM [13]	5	Public	93.94
CNN–SVM Model [33]	5	Private	86.69
Proposed Method	4	Private	95.18

**Table 5:** Compare fatigue classification accuracy (in %) based on the combination of denoising threshold function and classification algorithm.

Threshold Function	LSTM	CNN	SVM
Hard Threshold Function	88.71	86.78	82.90
Soft Threshold Function	91.33	89.84	87.63
Improved Threshold Function	95.18	92.72	90.30

## Data Availability

Access the data using the Google Cloud command line tools (please refer to the gsutil documentation for guidance): gsutil -m -u YOUR_PROJECT_ID cp -r gs://emgdb-1.0.0.physionet.org DESTINATION.

## Abbreviations

AT	anaerobic threshold
CNN	convolutional neural network
FF	false fatigue
FN	false non-fatigue
GA	gastrocnemius
IEMG	integrated electromyogram
LSTM	long short-term memory
MF	median frequency
MPF	mean power frequency
MSE	mean square error
MVC	maximum voluntary contraction
PSD	power spectral density
ReLU	rectified linear unit
RF	rectus femoris
RMS	root mean square
RMSE	root mean square error
RNN	recurrent neural network
sEMG	Surface electromyography
SNR	signal-to-noise ratio
SVM	support vector machine
TF	true fatigue
TN	true non-fatigue
VCO_2_	carbon dioxide production
VE	ventilation volume
VL	vastus lateralis
VM	vastus medialis
VO_2_	oxygen uptake
